# Case Report: A case of a multivisceral echinococcosis with atypical localization

**DOI:** 10.12688/f1000research.128900.1

**Published:** 2023-02-20

**Authors:** Rania Kaddoussi, Mohamed Jellazi, Abdelkader Mizouni, Ikram Chamtouri, Mabrouk Abdelaaly, Asma Migaou, Hager Ben Brahim, Nessrine Fahem, Ahmed Ben Saad, Sameh Joober, Saoussen Cheikhmhamed, Naceur Rouetbi

**Affiliations:** 1 Pneumology department, Fattouma Bourguiba hospital, monastir, tunisie, Tunisia; 2 Community medicine, Fattouma Bourguiba hospital, Monastir, tunisia, Tunisia; 3 Surgery department, Sahloul hospital, sousse, tunisia, Tunisia; 4 Cardiology department, Fattouma Bourguiba hospital, monastir, tunisia, Tunisia; 5 Radiology department, Fattouma Bourguiba hospital, monastir, tunisia, Tunisia; 6 Infectious disease department, Fattouma Bourguiba hospital, monastir, tunisia, Tunisia

**Keywords:** Hydtidosis, multi visceral, muscles

## Abstract

Hydatidosis is a pathology that is still common. The hydatid cyst commonly involves the liver and lung. Cases of multi visceral echinococcosis with atypical localization are rare. We report the case of a 53-year-old Tunisian farmer with a multiple organ hydatidosis that included 13 hydatid cysts: the lungs, the liver, the left heart ventricle, the left kidney, the abdomen cavity, muscles (psoas, adductors), and subcutaneous gluteal area. The majority of these cysts was already treated surgically, and some were due to be removed.

## Background

Hydatidosis is a human disease caused by the larval form of *Echinococcus* spp., which live in the gut of dogs, wild canines and other carnivorous animals. Humans become the accidental intermediate hosts by ingesting *Taenia* spp. eggs. *Echinococcus* spp. are endemic in many countries where sheep, dogs and man live in close contact.

All organs in the human body may be affected by hydatid disease. The hydatid cyst from *Echinococcus granulosus* commonly involves the liver and lung but may also be found in other unusual organs, including the brain, heart and bones. Hydatid cysts rupture into left-sided cardiac chambers may cause systemic emboli, and if ruptured into right-sided cardiac chambers may cause pulmonary emboli.

Cases of multi visceral echinococcosis with atypical localization are rare. We report here the case of a male with 13 hydatid cysts including the lung, the heart, the muscles, the liver, the kidney and abdomen cavity.

## Case report

This is the case of a 53-year-old maghribian male farmer. This patient, with no personal or family history, was referred to the pulmonology department, for the management of a pulmonary Aspergilloma on a sequelary lung cavity following lung surgery.

At the time of the encounter the patient had no subjective complaints, expect for right lower limb paresthesia. The physical exam showed no abnormalities.

**Figure 1.**
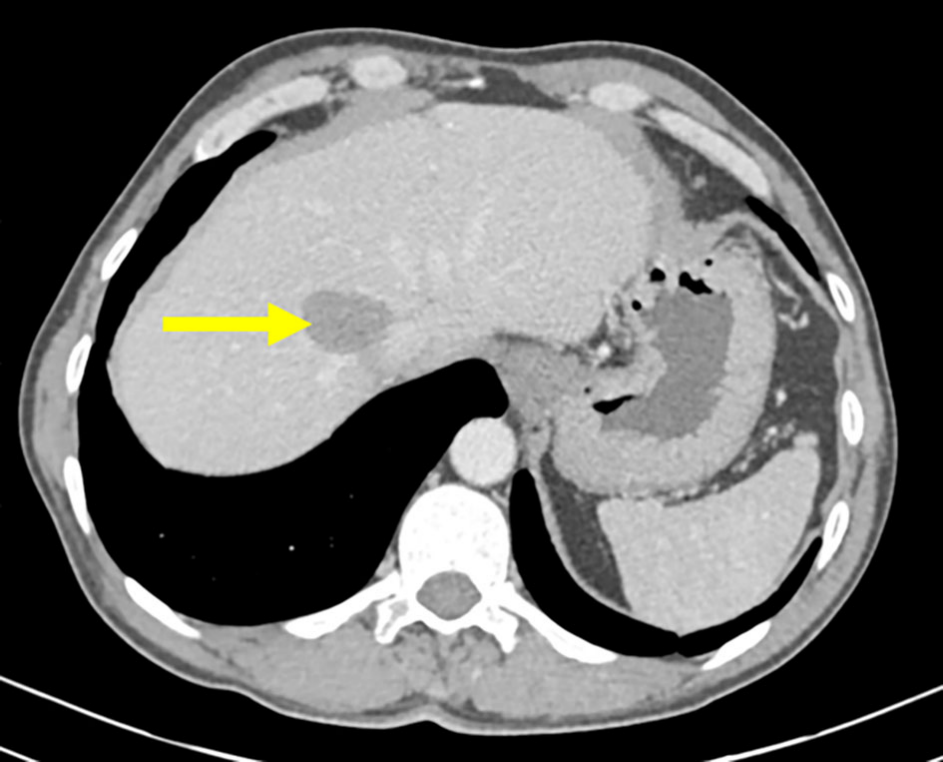
Axial chest scan passing through the liver showing a CE1 type hydatid cyst of segment 8 of the liver (yellow arrow).

**Figure 2.**
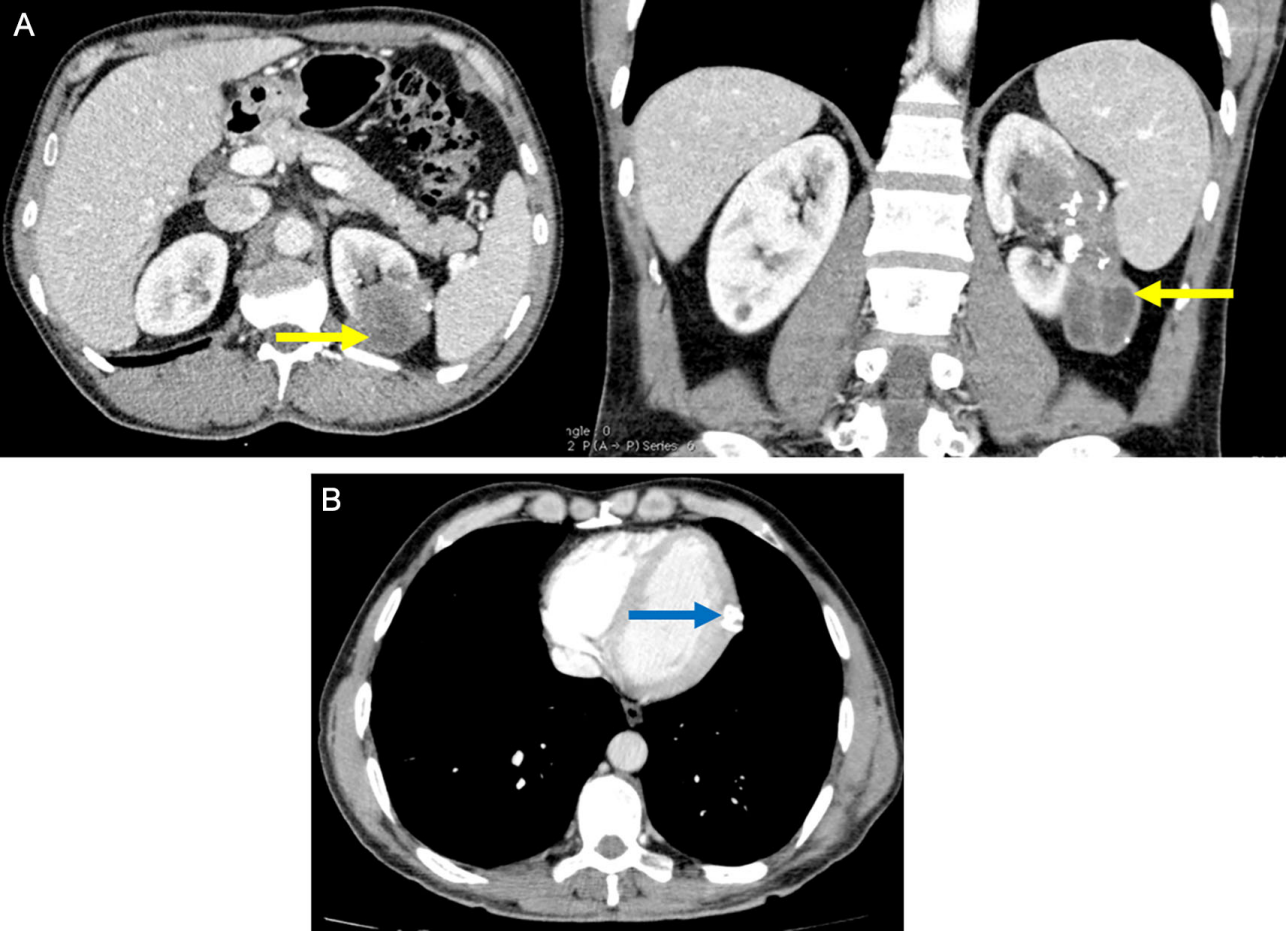
Axial computed tomography scans passing through the thorax in the mediastinal window and through the kidneys and coronal through the kidneys after injection of iodinated contrast product: Calcified hydatid cyst of the lateral wall of the left ventricle (blue arrow). Partially calcified multivesicular hydatid cyst with left endo and exo renal development (yellow arrow).

The patient was followed up in the abdominal surgery department for a multiple organ hydatidosis that included 13 hydatid cysts: the lungs, the liver ( Figure 1), the left heart ventricle, the left kidney ( Figure 2), the abdomen cavity, muscles (psoas, adductors), and subcutaneous gluteal area. The majority of these cysts was already treated surgically and some are still pending to be removed ( Table 1). The patient has received oral Albendazole 400 mg twice-daily for 2 years.

**Table 1.** Different types of organ involvement.

Organ	Location	Size	Treatment	Notes
Lungs	1) Right lungs	25×20 mm	Surgical	
	a) Left inferior lobe	N/A	Wedge resection	
	b) Left superior lobe	60 mm	Partially removed surgically	
Liver	Segment VIII	33×21 mm	Partially removed surgically	Between superior left and middle hepatic vein.
	Segment V	53×40 mm	Surgical removal+ Cholecystectomy	Tight adhesion with cystic bladder and right portal vein.
Kidney	Left kidney	10×80×79 mm	Surgical drainage	
Heart	Left ventricle	30 mm	Surgical (thoracotomy)	
Muscles	Psoas Adductor Gluteus	55×34 mm and 40×25 mm 20 mm	Surgical drainage	
Subcutaneous	Gluteal	15 mm	Surgical	
Abdomen cavity	Parieto colic gutter	60 mm	Surgical	
Abdomen cavity	Parietal peritonum	Bilobed	Surgical	

**Figure 3.**
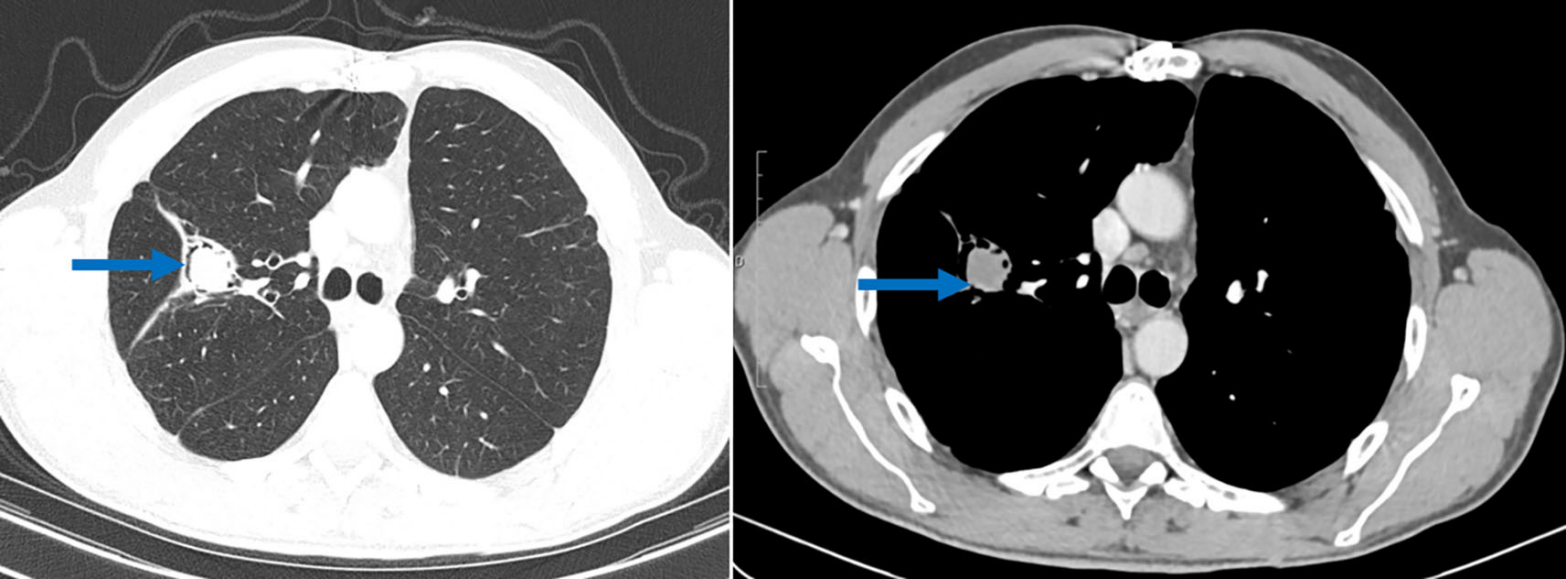
Axial chest scan passing through the thorax in the parenchymal window and after injection of iodinated contrast product showing a fluid-density endo-bronchial material in the right superior lobe of the lung with an “air crescent” sign: pulmonary aspergilloma (blue arrow).

During a regular post-surgical follow up, a scannographic image of a fluid-density endo-bronchial material in the right superior lobe (lung cavity sequelary to the previous cystectomy) of the lung separated from the cavity wall by an airspace (“air crescent” sign) was found, this image is typical of pulmonary aspergilloma ( Figure 3). Even though the Aspergillus serology showed doubtful results, the clinical context coupled to the CT scan findings were highly suggestive of a pulmonary aspergilloma. Blood work up showed high levels of IgE. A surgical resection of the cavity is programmed but not yet performed.

## Discussion

Hydatid cyst remains an important issue in Tunisian health that affects both humans and animals, especially in rural areas.

The annual incidence of hydatidosis is 11.3 per 100000 inhabitant. ^1^ Usually, hydatidosis presents as a single cyst usually of the liver or of the lungs, but in certain circumstances multiple organs may be affected. In our case we are reporting multiple atypical locations including heart, kidney, muscles and subcutaneous tissue. The conjunction of cysts in these locations simultaneously has not been described in the literature. We will not focus on the liver and the lung involvement given that they are a classic location of this parasitosis, and they are well commonly cited in the literature, instead we will discuss the other locations separately.

Heart involvement is uncommon and accounts for less than 0.5% of the cases, it is usually part of a disseminated infection. ^2^ This localization is potentially fatal without surgical treatment but fortunately with the improvement of surgical techniques, its morbidity has declined drastically. Our patient underwent open heart surgery to remove a left ventricular wall cyst without local recurrence and a post-surgical echocardiography without abnormalities.

The invasion of the myocardium usually occurs hematogeniously through the coronary arteries and since the majority of the population have a left dominant circulation, the left ventricle is the most commonly involved part of the heart (60%), ^3^ other explanation is the dissemination from the lungs either following a pulmonary vein rupture and migration of the cysts ^4^ or by a direct contact with hydatid cysts originating from the lung. ^5^

Renal involvement is also rare (2–3%) and it is usually associated to a disseminated disease, they are most commonly asymptomatic, like the case of our patient. The diagnosis was made by an abdominal CT which has a sensitivity of 98% to diagnose hydatid disease. ^6^

Psoas cysts is also uncommon, our patient presented with two psoas cysts, a finding never been described before in literature.

The patient has also presented with a 30-mm gluteal subcutaneous cyst, this involvement was described in rare cases in literature and usually the patient will have a painless palpable mass history of at least 3 months, and it is usually larger than 3 cm, which is the case in our patient. ^7^ Subcutaneous cysts tend to involve trunk and limb roots, possibly due to the rich vascularization and relatively less muscle activity in these areas. ^8^

Another intriguing finding in this case report is the discovery of an aspergilloma, on a lung cavity. Pulmonary aspergilloma occurs as a colonizer of pre-existing pulmonary cavity of any etiology such as sequelae tuberculosis, cavitary neoplasia or operated hydatid cyst and it is a saprophytic infection. ^9^ Aspergilloma has rarely been described in operated hydatid cyst cavities in immunocompetent patients. ^10^ For this patient, the aspergilloma was discovered two years after the lung surgery. A very similar case of a 56-year-old patient, who presented with an aspergilloma of the upper right lobe following cystectomy, have been described by M. El Hammoumi *et al*. ^10^

Despite the existence of multiple cysts, our patient is doing well with good tolerance and he is asymptomatic.

## Conclusion

Multiple hydatidosis is a rare condition which can endanger the vital and functional prognosis. Imaging is essential for the diagnosis and finds its place for the assessment of extension and detection of asymptomatic localization to ensure early treatment. Prevention remains the best treatment for hydatid cyst.

## Author contributions

NF, AM and AM actively involved in data collection and processing. RK and MJ were involved in manuscript preparation. HB, AB, SJ, SCH, NR, and FM were involved in manuscript reviewing. All authors have read and approved the manuscript.

## Consent

Written informed consent was received from the patient.

## Data Availability

All data underlying the results are available as part of the article and no additional source data are required.
