# Supplementary material for: Case Report: A case of a multivisceral echinococcosis with atypical localization
Source: F1000Res. 2023 Feb 20;12:193. [Version 1] doi: 10.12688/f1000research.128900.1 (PMC11470981; doi:10.12688/f1000research.128900.1)
Supplement: Peer review history

# Reviewer report: Reviewer response for version 1

**DOI:** 10.5256/f1000research.141537.r170271

**Published:** 2023-04-20

**Authors:** Mohamed Ali Chaouch

**Affiliations:** 1 Department of Visceral and Digestive Surgery, Monastir University Hospital, University of Monastir, Monastir, Tunisia

Thank you very much for the opportunity to review this paper. The subject is relevant and rare. However, some points of the manuscript should be re-addressed.

 1. Background:

- Add some reference for the different informations provided. The first reference is mentioned in the discussion section. 
- Switch "human disease" by "an anthropozoonosis" because hydatid cyst is not an only-human infection. 
- Add a sentence highlighting the interest of medical treatment in case of multiple hydatid cyst in addition to the surgical management.
- Add the aim of reporting this case to the end of the background.

2. Case presentation:

- Precisely which protocol of medical treatment was used?
- Mention if the patient presented any immune deficiency.

3. Discussion:

- Some citations were historical. Avoid citing articles published more than 20 years ago.
- Add the citation of the uncommon location for the psoas muscle.
- Add the citation of the different radiological signs suggesting the hydatid cyst origin of the lesions like the daugher cyst sign that is present in the kidney cyst in your case.
- Add some details concerning the management of these multiple cysts, like the importance of medical treatment and a radical surgery, avoiding conservative surgery, to reduce the reccurence rate. 

Are enough details provided of any physical examination and diagnostic tests, treatment given and outcomes?

Yes

Is the case presented with sufficient detail to be useful for other practitioners?

Yes

Is sufficient discussion included of the importance of the findings and their relevance to future understanding of disease processes, diagnosis or treatment?

Yes

Is the background of the case’s history and progression described in sufficient detail?

Partly

Reviewer Expertise:

I am a visceral and digestive surgeon in a pandemic country of hydatid cyst.

I confirm that I have read this submission and believe that I have an appropriate level of expertise to confirm that it is of an acceptable scientific standard, however I have significant reservations, as outlined above.

## References

[1] : A case report of primary multiple hydatid cysts of psoas muscle: An exceptional location. *IDCases* .2022;30: 10.1016/j.idcr.2022.e01637 10.1016/j.idcr.2022.e01637 PMC964340936388855

[2] : Daughter cyst sign in liver hydatid cyst. *J Parasit Dis* .2019;43(4) : 10.1007/s12639-019-01140-4 737-738 10.1007/s12639-019-01140-4 31749548 PMC6841845

# Reviewer report: Reviewer response for version 1

**DOI:** 10.5256/f1000research.141537.r164950

**Published:** 2023-03-14

**Authors:** Faisal Ahmed

**Affiliations:** 1 Urology Research Center, Al-Thora General Hospital, Department of Urology, School of Medicine, Ibb University of Medical Sciences, Ibb, Yemen

**General:**

- I strongly encourage the authors to have a manuscript reviewed by a fluent English speaker and writer to improve its language contents before resubmission.

**Abstract:**

- Please add a piece of important information regarding postoperative therapy and the outcome.

**Keywords:**

- Please add a case report in your keywords.

**Background:**

- It is inappropriate to have a background without a single reference. Please note that all statements must be supported by references.

**Case report:**

- This section should be structured according to the subsections included in the CARE Checklist of information to include when writing a case report. All the following subheadings should appear in this section, unless not applicable: Patient Information, Clinical Findings, Timeline of current episode, Diagnostic assessment, Diagnosis, Therapeutic interventions, and Follow-up and outcome of interventions.
- Please start with the primary concerns and symptoms of the patient. Then, medical, family, and psycho-social history including relevant genetic information, and finally, Relevant past interventions with outcomes.
- Significant History of contamination such as animal contact, water, etc., should be mentioned.
- Diagnostic testing such as CT scans, laboratory findings, etc., should be mentioned.
- Please add all details regarding surgical intervention.
- The follow-up period for your patient should be mentioned with details such as patient status, radiologic image result, recurrence, and recommended medications.

**Discussion: **

- For individual cases, it is advisable to describe how the case is rare/unusual and the educational/scientific merit of its publication, followed by an overview of the topic and a comparison of your case with similar cases described in the literature.
- There are some sentences that need citations. Please note that all statements must be supported by references.
- "The conjunction of cysts in these locations simultaneously has not been described in the literature."
  I suggest revising this sentence to "rarely reported in the literature" There are a few mentioned cases such as Cai *et al.* (2019 ^1^), Sachar *et al. *(2014 ^2^) and Dasbaksi *et al. *(2015 ^3^).
- Please discuss the factors below and compared them with your case:
  - A: Potential risk factors and predisposing factors of cystic echinococcosis.
  - B: Radiologic and laboratory tests.
  - C: Therapeutic options.
  - D: Recommended medication on follow-up and the duration.
  - E: Recurrence, and mortality.
  - F: The correlation between aspergilloma and echinococcosis infection.

**References: **

- I see you include old historical papers in references (1,4,5,7, and 9). It is not wrong; however, you fail to include recent articles in your review. Recent similar papers must be reviewed and compared to your case.
- There are some references not mentioned in your manuscript such as references 3 and 4 (all references must be numbered consecutively).

**Figures:**

- Figures 1 and 2: You can combine multiple images in a single panel. Please ensure you label the panel appropriately (A, B, C, etc.) in the image legend, and use the panel indicator to explain or describe the content of the image panel.

**Figure 2: **

- The photo of the kidney cyst should be reduced to one photo.
- Please add a photo of peritoneal and muscle cysts.

**Figure ligands:**

- Your ligands are so tall. Please reduce the legends to clearly describe the abnormalities.

**Table 1.**

- "Bilobed" please mention the exact size.
-  "Parieto colic gutter" please use the usual medical term.
- The treatment column did not provide any information and I suggest deleting it from the table and mentioning the details in your therapeutic intervention section.

Are enough details provided of any physical examination and diagnostic tests, treatment given and outcomes?

Partly

Is the case presented with sufficient detail to be useful for other practitioners?

Yes

Is sufficient discussion included of the importance of the findings and their relevance to future understanding of disease processes, diagnosis or treatment?

Partly

Is the background of the case’s history and progression described in sufficient detail?

Yes

Reviewer Expertise:

the discussion and case report sections need more revision.

I confirm that I have read this submission and believe that I have an appropriate level of expertise to state that I do not consider it to be of an acceptable scientific standard, for reasons outlined above.

## References

[1] : Case Report: Rare Presentation of Multivisceral Echinococcosis. *Am J Trop Med Hyg* .2019;100(5) : 10.4269/ajtmh.18-0673 1204-1207 10.4269/ajtmh.18-0673 30860020 PMC6493931

[2] : Uncommon locations and presentations of hydatid cyst. *Ann Med Health Sci Res* .2014;4(3) : 10.4103/2141-9248.133476 447-52 10.4103/2141-9248.133476 24971224 PMC4071749

[3] : A rare combination of hepatic and pericardial hydatid cyst and review of literature. *Int J Surg Case Rep* .2015;10: 10.1016/j.ijscr.2015.02.052 52-5 10.1016/j.ijscr.2015.02.052 25805610 PMC4429948
